# Natural Grass Cultivation Management Improves Apple Fruit Quality by Regulating Soil Mineral Nitrogen Content and Carbon–Nitrogen Metabolism

**DOI:** 10.3390/metabo13080925

**Published:** 2023-08-08

**Authors:** Bo Yu, Lixia Wang, Jiaqi Zhang, Deguo Lyu

**Affiliations:** College of Horticulture, Shenyang Agricultural University, Shenyang 110866, China; 2018200076@stu.syau.edu.cn (B.Y.); 2019200084@stu.syau.edu.cn (L.W.); 2021220304@stu.syau.edu.cn (J.Z.)

**Keywords:** apple, natural sod culture, photosynthesis, N metabolism, sugar metabolism

## Abstract

Orchard grass cultivation management is an effective measure to safeguard the sustainable development of the fruit industry in China. However, details of the influence of natural sod culture management on carbon (C)–nitrogen (N) nutrition of trees and fruit quality in Hanfu apple orchards are lacking. Therefore, a field experiment was conducted, which consisted of two treatments: clean tillage (CT) and natural grass cultivation (NG). Results revealed that NG treatment contributed to the increases in soil organic matter (SOM), total N, and soil NH_4_^+^-N at depths of 0–20 cm and 20–40 cm, while the soil NO_3_^−^-N concentration under NG treatment was significantly decreased at the same depth, within the range of 0–200 cm of the soil profile, compared with CT. NG treatment also significantly promoted leaf photosynthesis and enhanced leaf N and fruit sugar metabolism. The results of isotope labeling showed that NG treatment obviously elevated the ^13^C accumulation and distribution rate in fruits, as well as the ^15^N accumulation in the whole tree, whereas the ^15^N accumulation in fruits decreased. Furthermore, NG treatment significantly increased the fruit anthocyanin content. These results provide theoretical references for the feasibility of natural sod culture management to improve fruit quality in Hanfu apple orchards.

## 1. Introduction

Apples have been cultivated worldwide because of their unique health and economic value [1,2]. Appropriate N application is crucial for the growth and development of apples [3,4,5,6]. However, excessive and unreasonable application of N fertilizers (e.g., urea) has become a common phenomenon in apple orchards in China [7,8,9,10]. A large amount of unabsorbed N fertilizer is integrated into the soil N pool in the form of inorganic N or organic compounds [11]. Soil organic N can easily mineralize into ammonium N (NH_4_^+^-N) during the later stages of apple fruit expansion because of the effects of high temperature and rainy weather. Subsequently, NH_4_^+^-N in the soil can be easily converted to NO_3_^−^-N by nitrification, resulting in agricultural N pollution in orchards by increasing the risk of NO_3_^−^-N leaching into deep soil [12]. Moreover, apple trees prefer to utilize soil NO_3_^−^-N rather than other soil N sources [13]. A large amount of N absorbed through the root system during this period could easily lead to excessive N uptake by apple fruits due to the greater ability of fruits to accumulate N, resulting in excessive accumulation of N in fruits, which could have a negative influence on fruit quality, such as inhibiting the accumulation of photosynthates and the synthesis of anthocyanin in fruits [14,15], thereby decreasing the economic value of apple fruits.

The coordination and maintenance of C and N metabolisms are vital for strengthening crop growth and increasing the economic value of crops [16]. Fruit trees, including economically important crops, such as apples and pears, are primarily cultivated for fruit production. The economic value of these fruits is largely determined by their quality [17]. Substantial research has been conducted to explore effective solutions for improving fruit quality in orchards by coordinating tree C and N metabolism. For example, Tian et al. [2] indicated that the optimal use of Mg could elevate fruit quality by regulating C and N metabolism in leaves (source organs) and fruits (sink organs). Sha et al. [18] observed that the fruit quality could be significantly improved under PBZ (a plant growth regulator) treatment through altering the C and N nutrients in fruits. Orchard sod culture management, which has been proven to be beneficial for the high-quality and sustainable development of the Chinese fruit industry, is gradually being promoted in Chinese orchards. To date, the positive effects induced by orchard sod culture management on soil properties aspects have been proved [19,20,21,22]. Improving fruit quality is a core task in orchard management [17,23]. Recently, efforts have been made to explore the effects of grass cultivation management on fruit quality. Chen et al. [24] reported that grass mulching management could significantly improve fruit quality-related indices such as soluble sugar content. As reported by Han et al. [25], the application of green cover crops in pear orchards can improve the nutritive value of pear fruits. The meta-analysis results obtained by Ren et al. [26] showed that grass cultivation in orchards contributes to improved fruit quality. Fu et al. [27] and Muscas et al. [28] reported that orchard grass cultivation can reduce fruit acidity. However, some studies have found that the effects of grass cultivation management on fruit quality are not obvious [29]. There is large variability in the effects of orchard grass cultivation practices on fruit quality. Hanfu (2n = 34) has become the main apple variety cultivated in the cool areas of Northeast China because of its better cold resistance characteristics than other varieties of apples [30]. Currently, research on the application of orchard grass cultivation management in Hanfu apple orchards is mainly focused on soil aspects, such as elevating soil fertility, stimulating soil biological activities, and optimizing soil microbial community composition [31,32]. Details regarding the differences in Hanfu fruit quality between clean tillage practices and orchard grass cultivation management and their potential mechanisms are rarely reported, especially under natural sod culture conditions. Compared to artificially planted grass management, the implementation of natural sod culture management in orchards can effectively improve the ecological environment of orchards and promote the development of a virtuous cycle of orchard ecosystems. With its low investment and easy management, natural sod culture management in orchards has become a new direction for orchard soil management in China [33]. The coordination of C and N metabolism can directly or indirectly affect the formation of high-quality fruits [18]. Studying the differences in the C–N metabolism of Hanfu apple trees between clean tillage management and natural grass cultivation management is beneficial for exploring the effects of natural sod culture management on fruit quality to a certain extent.

In this study, ‘Hanfu’/GM256/*Malus baccata* Borkh. trees and the orchard inter-row soil were subjected to consecutive years of natural grass cultivation and clean tillage management. The findings aim to provide theoretical references for improving nitrogen nutrient utilization and enhancing fruit quality in apple orchards under natural sod culture management.

## 2. Materials and Methods

### 2.1. Study Sites Description

This study was performed in a Hanfu apple orchard located at Shenyang Agriculture University, Shenhe County, Shenyang City, Liaoning Province, Northeast China (123°56′ E, 42°23′ N). The mean annual temperature and annual precipitation of this region were 7.9 °C and 720 mm, respectively. Moreover, most of the precipitation in this region is concentrated between early August and September. The details of the soil properties are listed as follows: clay loam with 0.95% SOM, and the values of pH, available phosphorus, and available potassium were 5.75, 35.6 mg kg^−1^, and 76.2 mg kg^−1^, respectively.

### 2.2. Experimental Design and Sampling

In 2009, ‘Hanfu’/GM256/*Malus baccata* Borkh. seedlings of similar growth were planted, and the cultivation density was 2.0 m × 4.0 m. Two inter-row soil management systems were adopted in this study, named clean tillage management (CT) and natural grass cultivation (NG) management. Each treatment (management) consisted of three replicates, each with an area of 200 m^2^ and 30 apple trees. Prior to establishing the two soil managements (2009–2014), the soil of each replicate received the same soil management, with the understory cleaned periodically. The field experiments were conducted in 2015. Clean tillage (CT) indicates the soil surface was tilled and the weeds were removed in a timely and regular manner. For the natural grass cultivation (NG) management, the soil in the tree rows was managed as CT, while the soil in the inter-row was grown with natural weeds such as *Polygonum aviculare* Linn. and *Digitaria sanguinalis* (Linn.) Scop. The inter-row grass was mown three times a year, and the plant residues were mulched in situ. Moreover, trees under different treatments were trained as slender spindles, and the fertilization scheme of each tree remained consistent between the CT and NG treatments. In total, 340 g normal urea (CO(NH_2_)_2_), 210 g ammonium phosphate ((NH_4_)_2_HPO_4_), and 120 g potassium sulfate (K_2_SO_4_) were applied to each tree from 2015 to 2021. Half of the fertilizers were applied at the germination stage, and the remaining were applied as fruit-setting fertilizers. On a circular surface with a radius of about 150 cm, 5 points were evenly selected, and fertilizer pits with a depth of 20 cm and a diameter of 20 cm were dug. In addition, other relevant agronomic practices (such as pruning and irrigation) were consistent.

^15^N labeling fertilization was conducted in 2022. Twelve trees from each treatment group were selected and used as the test material. To exclude the ^15^N and ^13^C natural abundances in tree organs influenced by different soil management practices, the 12 trees mentioned above were divided into two groups with six trees per group and were named the normal group and labeling group. The fertilization details of the normal group were as follows: the amounts of urea, ammonium phosphate, and potassium sulfate applied to each tree were 340, 210, and 120 g, respectively. The fertilization details of the labeled group were similar to those of the normal group, except that 340 g of normal urea was replaced with 10 g ^15^N-urea (10.14%, abundance) and 330 g of normal urea.

Following the steps described by Wang et al. [14], ^13^C labeling was performed 147 days after blooming. Briefly, labeling chambers made of Mylar plastic bags and brackets were prepared to cover and seal the trees of the label group for each treatment. Each tree corresponds to a single labeling chamber. A beaker containing 10 g of Ba^13^CO_3_ together with fans and reduced iron powder was placed in the chamber. After turning on the fan and sealing the chamber, ^13^C pulse labeling was performed. To maintain the ^13^CO_2_ concentration, we injected into the beaker every 30 min. The concentration of hydrochloric acid used in ^13^C pulse labeling was set to 1 mol L^−1^. The ^13^C pulse-labeling process lasted for 4 h.

Soil samples were obtained using a soil drill 150 d after blooming. Within a range of 0–300 cm of the soil profile, soil samples were collected at 20 cm intervals in the vertical direction of each soil sampling point. Twelve soil extraction sites were evenly distributed within the canopy projection and were occupied by a tree. After removing large rocks and plant residues, 12 soil samples collected from the same depth were considered as one replicate and homogeneously mixed. Soil samples harvested from the normal group were used to measure SOM, soil total N, and soil mineral N (NH_4_^+^ and NO_3_^−^) content. Then, 72 h after the end of ^13^C pulse labeling (150 days after blooming), all trees in the labeling group were subjected to destructive sampling.

### 2.3. Experimental Index Measurements

#### 2.3.1. Levels of SOM, TN, and the Contents Soil NO_3_^−^-N and NH_4_^+^-N

Following the method described by Bremner and Jenkinson [34], the SOM value was confirmed, and the method reported by Bao [35] was employed to determine the level of total N. The method described by Duan et al. [36] was used to determine the contents of NO_3_^−^-N and NH_4_^+^-N in the soil profile.

#### 2.3.2. Photosynthetic Parameter

The values of leaf net photosynthetic rate (*P*_n_) and stomatal conductance (*G*_s_) were confirmed at 120, 135, and 150 days after blooming using an LI-6400XT portable photosynthesis system (LI-Cor, Lincoln, NE, USA). The measurements started at 9:00 and ended at 11:00. The light-saturation point was set to 1200 μmol (photon) m^−2^ s^−1^ to measure net photosynthetic rate, and the CO_2_ concentration was 400 μmol (CO_2_) mol^−1^. During the same period, a pulse-modulated chlorophyll fluorescence meter (PAM 2500; Walz, Germany) was used to determine the chlorophyll fluorescence parameters of leaves. Leaves were collected at the end of the measurement of photosynthetic parameters and brought to the laboratory. The steps concluded by Liu et al. [37] were used for the analysis of Ribulose-1,5-biphosphate carboxylase-oxygenase (Rubisco).

#### 2.3.3. Levels of Sorbitol, Sucrose, Fructose, and Glucose

The method presented by Ma et al. [38] was adopted in this experiment to prepare the measurement. The levels of sorbitol, sucrose, fructose, and glucose in leaves and fruits were determined by filtration using liquid chromatography methods by Filip et al. [39].

#### 2.3.4. Enzyme Activities Determination

Sorbitol 6-phosphate dehydrogenase (S6PDH) activity was measured according to the methods presented by Berüter [40]. Sucrose synthase (SS) and sucrose phosphate synthase (SPS) activities were measured as by Hu et al. [41].

The method reported by Tian et al. [2] was used to prepare the enzyme solution, which was then dialyzed and used to determine sorbitol dehydrogenase (SDH), sorbitol oxidase (SOX), sucrose synthase decomposition direction (SS-c), acid invertase (AI), and neutral invertase (NI) activities. Subsequently, the methods described by Rufly and Huber [42] and Yamaki and Asakura [43] were adopted to determine SDH and SOX, respectively. SS-c activity was measured as described by Huber [44]. The AI and NI were determined following the steps described by Merlo and Passera [45]. In addition, the activities of N-metabolism-related enzymes were investigated. Briefly, the methods presented by Ding et al. [46] and Seith et al. [47] were used to analyze the nitrate reductase (NR) and nitrite reductase (NiR) activities, respectively. The glutamine synthetase (GS) and glutamate synthase (GOGAT) activities were measured as described by Hu et al. [48].

#### 2.3.5. ^15^N and ^13^C Isotope Analysis

The trees in the labeling group for each treatment were divided into five parts, from bottom to top, which contained the roots, trunks, perennial branches, annual branches, leaves, and fruits. Preparation before the determination of ^15^N and ^13^C abundance and the calculation of ^13^C and ^15^N abundance followed the method reported by Xu et al. [49]. Subsequently, the samples prepared for measuring ^15^N abundance were transferred to the laboratory, and measurements were performed using a MAT-251-Stable Isotope Ratio Mass Spectrometer. The samples used for ^13^C abundance determination were analyzed using a DELTAVplus XP advantage isotope ratio mass spectrometer. The computational formulae for ^15^N and ^13^C-related indexes are as follows:

Calculation of ^15^N

Ndff (%) = [(abundance of 15N in plant − natural abundance of 15N)/(abundance of 15N in fertilizer − natural abundance of 15N)] × 100%(1)

^15^N absorbed by each organ = Ndff (%) × total N content (mg) (2)

Calculation of ^13^C

Abundance of ^13^C: F*_i_* (%) = [(δ^13^C + 1000) × R_PBD_]/[(δ^13^C + 1000) × R_PBD_ + 1000] (3)

R_PBD_ is a constant value that represents the standard ratio of carbon isotopes. The value of R_PBD_ is set to 0.0112372.

C content of each organ:

C*_i_* = organ dry matter (g) × organ total carbon content (%) (4)

Content of ^13^C in each organ:

^13^C*_i_* (mg) = [C*_i_* × (F*_i_* − F*_nl_*)/100] × 1000 (5)

The value of F*_nl_* represents the ^13^C natural abundance of each organ:

^13^C partitioning rate: ^13^C (%) = (^13^C*_i_*/^13^C _net absorption_) × 100% (6)

#### 2.3.6. Fruit Quality Indexes Measurement

A Vernier caliper transverse was used to measure the fruit and longitudinal diameters. Following the method reported by Zheng et al. [50], we analyzed the anthocyanin concentration. Before calculating the sugar–acid ratio, the methods described by Liu et al. [51] and Nie et al. [52] were adopted to measure the soluble sugar and titratable acid contents, respectively.

#### 2.3.7. RNA Isolation and qRT-PCR Analysis

In this study, the relative expression of genes involved in sorbitol and sucrose transport was analyzed. First, following the guidelines of RNAiso Plus (Takara, Otsu, Shiga, Japan), total RNA was extracted from the samples. Subsequently, the concentration and purity of total RNA from the samples were determined. ReverTra Ace^®^ qPCR RT Master Mix with gDNA Remover (TOYOBO, Osaka, Japan) was used to synthesize cDNA. Relative gene expression was analyzed by RT-qPCR on a LightCycler 96 (Roche, Basel, Switzerland) using TranStart Top Green qPCR SuperMix (TransGen Biotech, Beijing, China). The expression was normalized according to the method described by Xu et al. [49]. The primers used are listed in Supplementary Table S1. Each treatment consisted of three technical and three biological replicates.

### 2.4. Data Collection and Statistical Analysis

The data for the indices in this experiment were collected in Microsoft Excel and analyzed using SPSS (version 21.0; SPSS Inc., Chicago, IL, USA). The significance of differences between the two groups (treatments) was analyzed using a *t*-test (Student’s *t*-test). * represents *p* < 0.05, ** represents *p* < 0.01.

## 3. Results

### 3.1. Level of SOM, TN, and Soil Mineral N (NH_4_^+^-N and NO_3_^−^-N)

#### 3.1.1. SOM and TN

**Figure 1:**
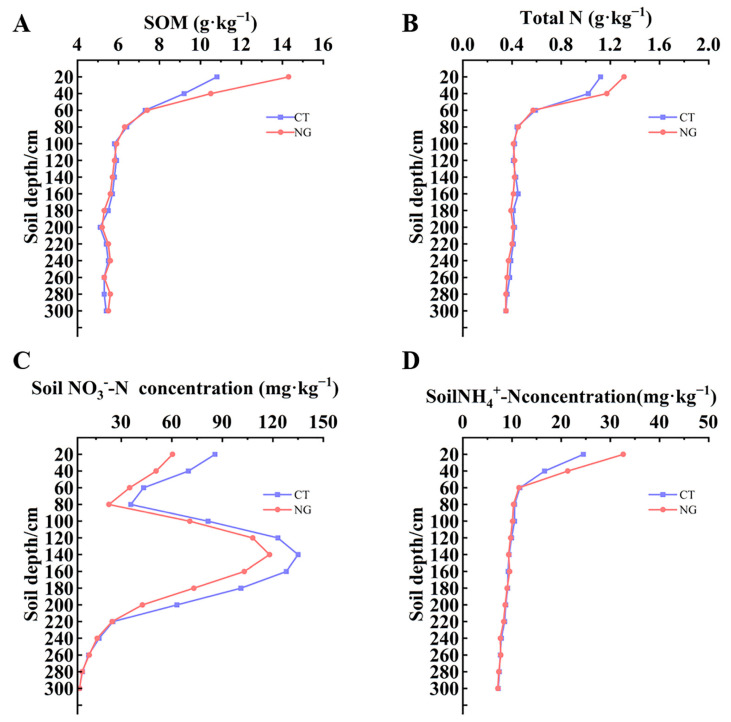
SOM (**A**), soil total N (**B**) as well as soil NO_3_^−^-N (**C**) and NH_4_^+^-N (**D**) concentrations at 0~300 cm under between CT and NG treatments. CT: clean tillage; NG: natural grass cultivation.

Different soil management methods significantly affected the SOM levels. As shown in Figure 1A, NG management in the orchard mainly affected the SOM content at 0–40 cm depth. The values of SOM under the NG treatment at 0–20 cm and 20–40 cm were 14.3 g kg^−1^ and 10.50 g kg^−1^, respectively, which increased by 32.41% and 14.13%, respectively, compared to CT. However, no significant difference was observed in the SOM content of the 40–300 cm soil layer between the CT and NG treatment groups.

As presented in Figure 1B, the values of soil total N under the NG treatment at 0–20 cm and 20–40 cm were 1.31 g kg^−1^ and 1.17 g kg^−1^, respectively, which increased by 16.96% and 14.71%, respectively, compared to CT. Similar to the changes in SOM, no significant difference existed in the soil total N content in the 40–300 cm soil layer between the CT and NG treatments.

#### 3.1.2. Soil Mineral N (NH_4_^+^-N and NO_3_^−^-N)

Regardless of the treatment, at a soil depth of 0–80 cm, the soil NO_3_^−^-N concentrations decreased with increasing soil depth, whereas a trend of increasing and then decreasing with increasing depth of the soil layer was observed at 80–260 cm. In the 200–300 cm soil layer, no significant fluctuations in soil NO_3_^−^-N concentration with the increasing depth of the soil layer. Within the range of 0–200 cm of the soil profile, the soil NO_3_^−^-N concentration treated with NG was obviously lower than that with CT at the same depth (Figure 1C). The concentrations of NH_4_^+^-N at 0–20 cm and 20–40 cm depths under the NG treatment were significantly higher than those under CT. However, at the same depth, no obvious differences were observed between the CT and NG treatments within the range of 40–300 cm in the soil profile (Figure 1D).

### 3.2. Plant C–N Nutrition

#### 3.2.1. Photosynthetic Parameter

**Figure 2:**
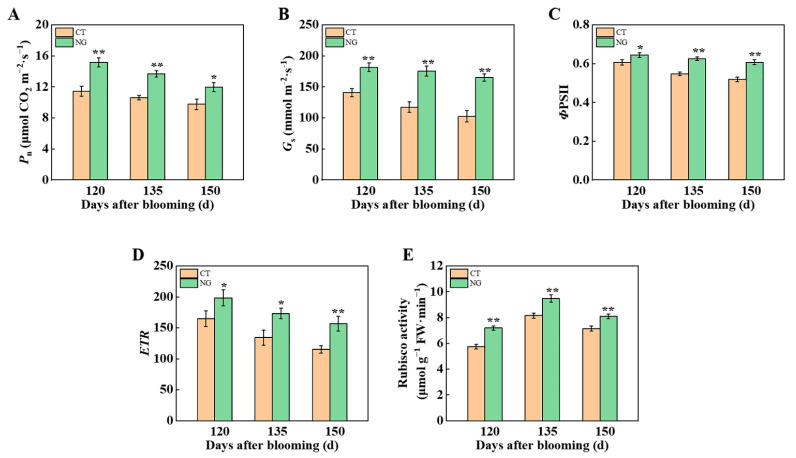
*P*_n_ (**A**), *G*_s_ (**B**), *Φ*PSII (**C**), *ETR* (**D**) and Rubisco activity (**E**) between CT and NG treatments. CT: clean tillage; NG: natural grass cultivation. * represents *p* < 0.05, ** represents *p* < 0.01.

As shown in Figure 2, the NG treatment enhanced the photosynthetic ability of the leaves. During the same sampling period, *P*_n_ and *G*_s_ of leaves treated with NG were greater than those of leaves treated with CT (Figure 2A,B). Compared to CT, NG treatment also significantly elevated the levels of *Φ*PSII (quantum yield of photosystem II electron transport) and *ETR* (electron transport rate) (Figure 2C,D). In addition, leaves in the NG treatment showed significantly higher RuBisCO activity than those in the CT treatment. The RuBisCO activity under NG treatment increased by 25.48%, 16.18%, and 13.29%, respectively, compared to the CT (Figure 2E).

#### 3.2.2. C Metabolism-Related Enzyme Activities in Leaves and Fruits

**Figure 3:**
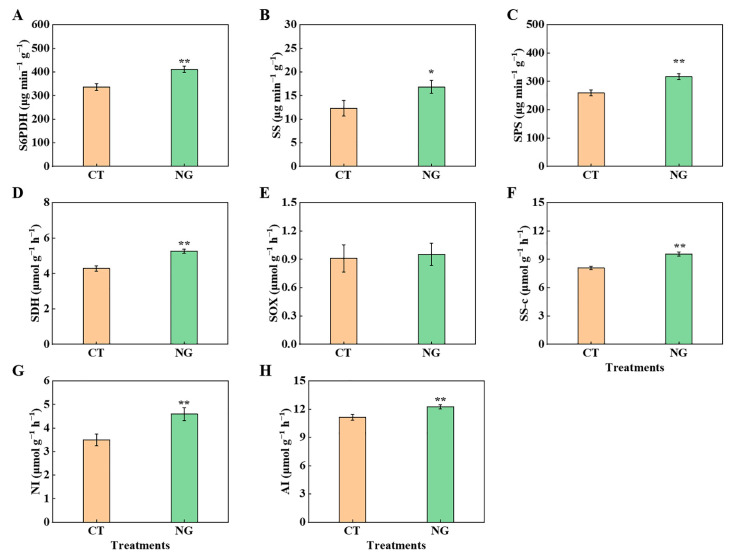
S6PDH (**A**), SS (**B**), SPS (**C**) enzyme activities in leaves and SDH (**D**), SOX (**E**), SS-c (**F**), NI (**G**), AI (**H**) in fruits. * represents *p* < 0.05, ** represents *p* < 0.01.

Differences in the C metabolism-related enzyme activities in leaves and fruits between CT and NG are shown in Figure 3. The activities of SDH, SS-c, AI, and NI in fruits were 1.23, 1.18, 1.10, and 1.32 times higher than those in CT, respectively, and no noticeable difference in SOX activity was found in fruits. Moreover, we also measured the leaves’ S6PDH, SPS, and SS activities. The values of them were elevated by NG treatment, which were increased by 22.03%, 22.03%, and 36.75%, respectively, compared to the CT.

#### 3.2.3. Concentrations of Sucrose, Sorbitol, Fructose, and Glucose in Leaves and Fruits

**Figure 4:**
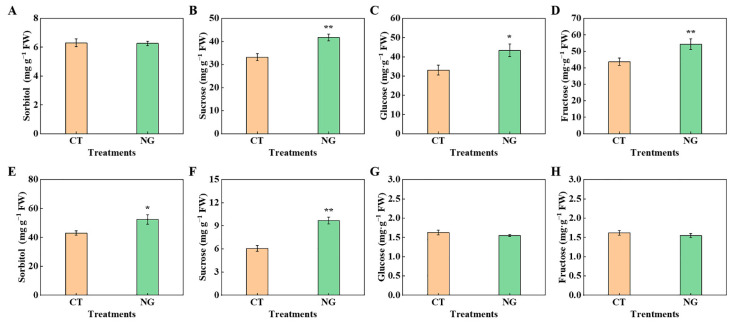
Fruits’ sorbitol (**A**), sucrose (**B**), glucose (**C**), fructose (**D**), and leaves’ sorbitol (**E**), sucrose (**F**), glucose (**G**), and fructose (**H**) concentrations between CT and NG treatments. * represents *p* < 0.05, ** represents *p* < 0.01.

NG treatment significantly elevated the sorbitol and sucrose contents of the leaves, compared to the CT. The fructose, glucose, and sucrose contents in fruits treated with NG were also significantly higher than those in CT, by 24.66%, 31.25%, and 25.67%, respectively. Moreover, there was no obvious difference in the concentrations of fructose and glucose in the leaves, or sorbitol in the fruits between the CT and NG treatments (Figure 4).

#### 3.2.4. N Metabolism-Related Enzyme Activities in Leaves

**Figure 5:**
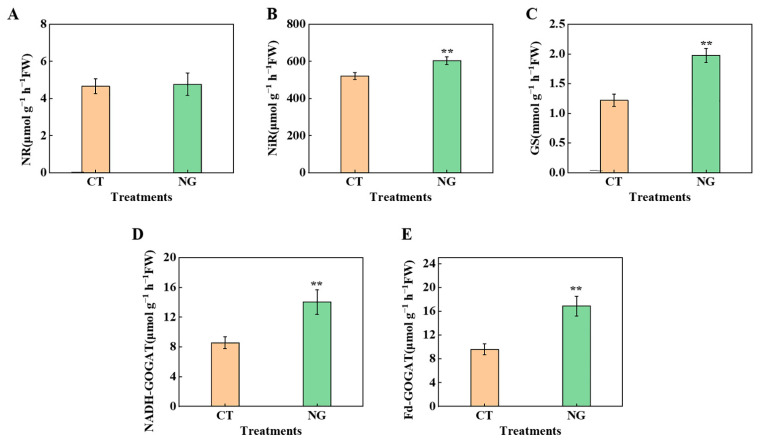
Leaves’ N metabolism enzyme activities between CT and NG treatments. NR (**A**), NiR (**B**), GS (**C**), NADH-GOGAT (**D**), and Fd-GOGAT (**E**). ** represents *p* < 0.01.

As shown in Figure 5, under NG treatment, the values of NiR, GS, NADH-GOGAT, and Fd-GOGAT were significantly elevated, with values of 15.92%, 61.48%, 63.71%, and 75.73%, respectively, compared with CT. However, no obvious difference was observed in NR activity between the CT and NG treatments.

#### 3.2.5. ^13^C distribution Rate and ^13^C Accumulation in Organs

**Figure 6:**
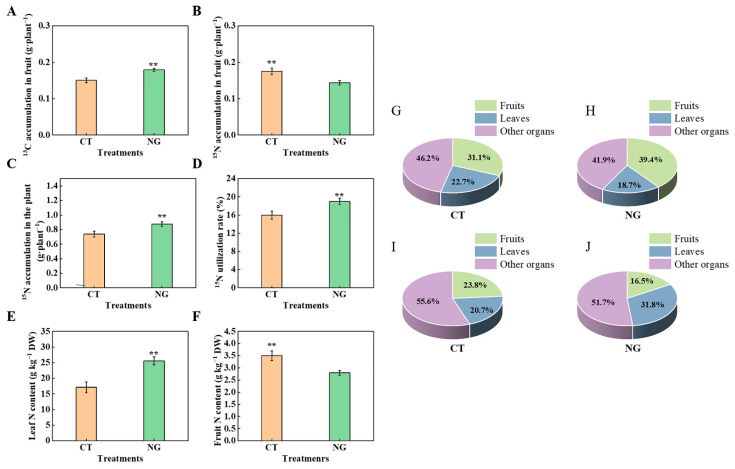
^13^C accumulation in fruits (**A**), ^15^N accumulation in fruits (**B**), ^15^N accumulation in the whole plant (**C**), ^15^N utilization rate (**D**), leaves N content (**E**), fruits N contents (**F**) as well as ^13^C (**G**,**H**) and ^15^N distribution ratio (**I**,**J**) under various treatments. ** represents *p* < 0.01.

In this study, the difference in ^13^C distribution rate between CT and NG in different organs was analyzed using ^13^C isotope labeling. As illustrated in Figure 6G,H, under the NG treatment, the distribution of ^13^C in the fruits increased significantly, while that in the leaves decreased. No significant differences were observed in other organs (perennial branches, annual branches, roots, and trunk) between the CT and NG treatments. In addition, we further analyzed the ^13^C accumulation in fruits and found that NG treatment significantly increased fruit ^13^C accumulation compared to CT, which was 20.00% higher than that in the CT treatment (Figure 6A).

#### 3.2.6. ^15^N Accumulation and ^15^N Distribution Rate

According to the results of ^15^N labeling, compared with CT, ^15^N accumulation in the whole plant increased by 18.92%, whereas ^15^N accumulation in fruits decreased by 22.22% (Figure 6C,D). We further analyzed the differences in the organ ^15^N distribution rate between the CT and NG treatments. Compared with CT, the leaf ^15^N distribution rate was significantly elevated under the NG treatment. The fruit ^15^N distribution rate showed the opposite tendency, showing a significant decrease under the NG treatment compared with CT (Figure 6I,J).

#### 3.2.7. Gene Expression of Sugar Transporter

**Figure 7:**
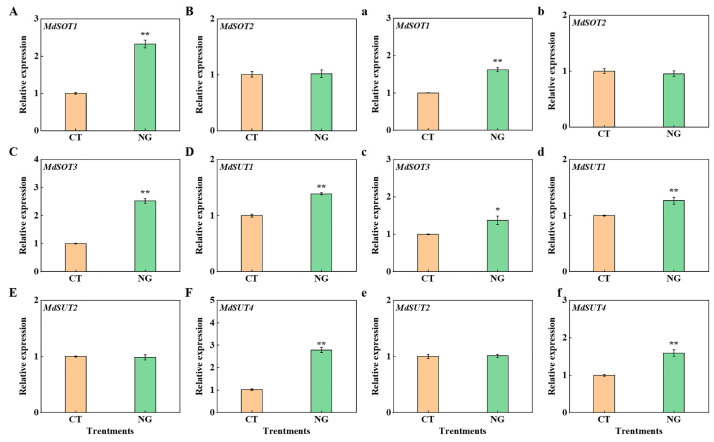
Genes relative expression in fruit flesh (**A**–**F**) and stalk (**a**–**f**) between CT and NG treatments. * represents *p* < 0.05, ** represents *p* < 0.01.

As shown in Figure 7, the relative expression of *MdSOT1*, *MdSOT3*, *MdSUT1*, and *MdSUT4* in fruit flesh and stalk was obviously promoted under the NG treatment compared to the CT treatment.

### 3.3. Fruit Quality

**Figure 8:**
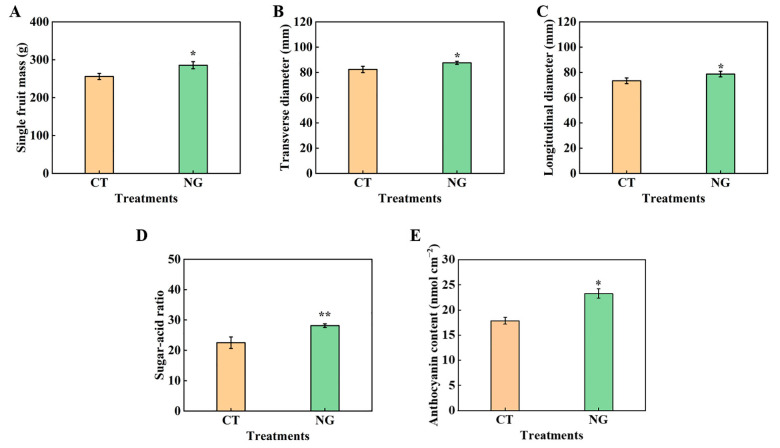
Single fruit weight (**A**), transverse diameter (**B**), longitudinal diameter (**C**), sugar–acid ratio (**D**) as well as anthocyanin content (**E**) between CT and NG treatments. * represents *p* < 0.05, ** represents *p* < 0.01.

As presented in Figure 8, under NG treatment, the single fruit weight and the transverse and longitudinal diameters of the apples increased significantly. Moreover, compared with CT, NG management significantly elevated the anthocyanin content in the fruits and maintained an appropriate sugar–acid ratio.

## 4. Discussion

### 4.1. Natural Sod Culture Management Alters N Absorption of Trees and Reduces the Risk of Soil Nitrate Leaching

Consistent with frequently observed increases in soil total N and SOM content in diverse climatic zones [21,53], our results showed that orchard natural grass cultivation (NG) management significantly elevated the levels of SOM and TN compared to clean tillage (CT). This indicates an improvement in soil fertility under natural grass cultivation conditions. After exogenous N fertilizer (such as urea) is applied to the soil, urea is rapidly hydrolyzed to NH_4_^+^ through the action of urease and rapidly participates in soil N pools [11]. Soil NH_4_^+^-N is readily adsorbed and fixed by soil organic matter and colloidal particles [14]. It has been widely acknowledged that long-term grass cultivation management could significantly elevate the levels of SOM and colloidal particles [22]. In this study, we found that the soil NH_4_^+^-N concentrations at depths of 0–20 cm and 20–40 cm were obviously elevated under the NG treatment. Notably, we also observed that the soil NH_4_^+^-N concentrations at 0–20 cm and 20–40 cm were higher than those within the 40–300 cm range of the soil profile, regardless of soil management. This observation may be closely related to the variations in SOM and colloidal particle content with depth in the soil pool. Nitrate N pollutes surface water and groundwater through surface runoff and leaching losses, as well as the atmosphere through denitrification, thus resulting in agricultural N pollution [14]. The results described by Garcia-Diaz et al. [54] in a vineyard indicated that spontaneous vegetation cover management could effectively reduce the risk of soil nitrate-N loss compared to conventional tillage. Stork and Jerie [55] reported that apricot orchards covered with cocksfoot effectively slowed nitrate leaching through N fixation. Moreover, the results presented by Wang et al. [56] revealed that the reduction in NO_3_^−^-N concentration in the soil profile would be favorable for decreasing the risk of NO_3_^−^-N leaching in the soil. In this study, we found that the soil NO_3_^−^-N concentrations at depths of 0–200 cm were reduced under NG treatment. This reduction may be attributed to the natural grass vegetation grown in the orchard inter-row soil, which can absorb part of the NO_3_^−^-N present in the 0–20 cm soil profile, and then reduce the risk of nitrate leaching to the deeper soil profile.

N is the primary focus of precise nutrient management in orchards [57,58]. Consistent with the results obtained by Peng et al. [59], the ^15^N labeling results showed that ^15^N accumulation in the whole tree and ^15^NUE were both elevated under NG treatment, indicating that the N absorption ability of apple trees was enhanced under NG treatment. Numerous studies have reported that orchard grass cultivation management can alter and optimize soil structure by modifying the composition of soil aggregates and enhancing soil nutrient availability through accelerated nutrient cycling [60,61]. Moreover, Zhou et al. [32] indicated that the inter-row grasses mowing management could affect soil microbial community structure and function by changing root exudate composition, and then promotes soil N cycling process, which could also affect soil N supply potential. These changes can significantly affect plant root growth and nutrient absorption capacity to a certain extent. Therefore, the strengthening of the N absorption ability of trees could be closely related to improvements conferred by grass cultivation management in some aspects of the soil. Moreover, the reduction in soil NO_3_^−^-N leaching risk, as indicated by the decrease in soil NO_3_^−^-N concentrations at depths of 0–60 cm under NG treatment, could also be attributed to the stronger N absorption ability of apple tree roots under NG treatment. The NO_3_^−^-N absorbed by roots from the soil pool needs to undergo various physiological and biochemical processes under the reaction of a series of N metabolism-related enzymes before it can be converted into organic N for its own use [10]. The efficient functioning of N metabolic processes under the actions of N metabolism related enzymes in leaves (the main site of photosynthesis in fruit trees) is of great importance for high-quality fruit formation because of the close relationship between N metabolism and photosynthesis, as well as photosynthesis-mediated carbon fixation. Therefore, we measured leaf NR, NiR, GS, and GOGAT activities and found that NG treatment significantly increased leaf NiR, GS, and GOGAT activities, indicating that N metabolism in leaves was enhanced under NG treatment. Additionally, there was no obvious difference in NR activity between the CT and NG treatment groups. A similar result was observed in ‘Red Fuji’ apple under various magnesium (Mg) conditions performed by Tian et al. [2]. Apart from the obvious differences in ^15^NUE observed from ^15^N labeling experiment, we also found that the leaf ^15^N distribution rate increased significantly with NG application. This might be because the enhancement of N metabolism in NG-treated leaves strengthened the leaf N demand. Wang et al. [8] showed that increased leaf N demand could lead to a reduction in N distribution and accumulation in fruits, which is beneficial for the elevation of sugar levels to a certain extent by avoiding excessive N accumulation in fruits. Similar results were observed in the present study.

### 4.2. Natural Sod Culture Management Alters Tree C–N Nutrition and Improves Fruit Quality

Fruit quality directly depends on photosynthesis-mediated C (CO_2_) fixation and the transport of photoassimilates from the leaves to the fruit [62]. In this experiment, the differences in *P*_n_ and *G*_s_ of the leaves between CT and NG were analyzed. Compared with the CT, the values of leaves’ *P*_n_ and *G*_s_ were both significantly elevated under the NG treatment. As described by Lawlor and Cornic [63], the synthesis of photosynthetic assimilates in leaves is realized by using CO_2_ as raw material. A higher *G*_s_ under the NG treatment indicates that the leaves’ CO_2_ absorption ability was elevated, which could explain the difference in fruit ^13^C accumulation between the CT and NG treatments. Furthermore, we also found that NG treatment significantly increased *ETR* in leaves, and the elevation of *ETR* in leaves treated with NG indicated an increase in the PSII electron transfer efficiency. Furthermore, the activity of Rubisco treated with NG was obviously greater than that of Rubisco treated with CT, indicating that NG treatment improved the capacity of photosynthetic assimilate synthesis and ensured the source strength of the leaves. Moreover, the N absorption ability of the roots is closely related to leaf photosynthesis [64]. Therefore, the enhancement of leaf photosynthesis caused by the NG treatment could also explain why the ^15^N accumulation of the whole tree under the NG treatment was higher than that under the CT treatment.

The synthesis of photosynthetic assimilates and their targeted transportation from leaves to fruits are inextricably linked to sugar accumulation in apples [65,66]. As a perennial woody plant of the Rosaceae family, the main photosynthates of apples are sorbitol and sucrose, accounting for 80% and 20%, respectively [67]. We found that the NG treatment significantly elevated the sorbitol and sucrose contents of the leaves. The reason might be due to the increases in the activities of S6PDH, SPS, and SS. The elevation of leaves’ sorbitol and sucrose contents indicated that the source strength was elevated under the NG treatment. Enhanced source strength is of great importance for the output of photosynthates [2], which could explain why the leaf ^13^C distribution rate under NG management decreased. Moreover, the elevation of the relative expression of *MdSOT*s and *MdSUT*s in in fruits under NG treatment provides additional evidence of enhanced sugar transport. Zhang et al. [68] reported that the enhancement of sorbitol and sucrose decomposition-related enzyme activities had a significant positive effect on sugar accumulation in fruits. In the present study, under NG management, the activities of SS-c, AI, NI, and SDH in fruits were elevated, indicating that NG management promoted sorbitol and sucrose decomposition, thereby increasing fruit sink strength. There was no significant difference in SOX activity between the CT and NG treatment groups. This may be related to the bound form and the lower activity of SOX [68]. The sorbitol and sucrose decomposition of fruits under the reaction of sugar-metabolism-related enzymes could not only result in a decrease in the vacuole osmotic potential but also elevate the concentration gradient of sorbitol in both source and sink organs, which could enhance the efficiency of sorbitol transport from leaf to fruit [69]. Consistent with the findings reported by Tian et al. [2], the sucrose content in leaves and fruits indicated that sucrose transport from leaves to fruits was an inverse concentration-gradient process. The elevation of sucrose transport from leaves to fruits in the NG treatment may be related to changes in energy and sucrose transporters.

The late stage of fruit expansion is a critical period that affects apple quality. However, excessive accumulation of N in apple trees often occurs during this period because of the high soil NO_3_^−^-N absorption. It has been widely acknowledged that excessive N accumulation in fruits negatively influences the formation of high-quality apple fruits [70]. The results of ^15^N and ^13^C labeling showed that NG management decreased ^15^N accumulation in the fruits. However, the ^13^C accumulation and ^13^C distribution rates of fruits were significantly elevated under the NG treatment, which was consistent with the tendency of sugar content elevation in fruits (mentioned above). As presented by Wang et al. [14], high fruit N accumulation can decrease sugar decomposition-related enzyme activities and inhibit the transportation of photosynthates to leaves and fruits. Therefore, the elevation of sugar decomposition-related enzyme activities and the enhancement of sugar transport from leaves to fruits (as mentioned above) could be closely related to the decrease in ^15^N accumulation in fruits. Compared to fruit yield, fruit farmers are becoming aware of the importance of fruit quality. Consumers prefer to purchase apples with good appearance and taste. In this study, under NG treatment, the single fruit weight and fruit transverse and longitudinal diameters increased significantly, indicating that the sink zone increased, which could be conducive to sugar accumulation. Moreover, we found that NG management maintained an appropriate sugar–acid ratio and increased anthocyanin content. This may be closely related to the elevated sugar content in the fruits [15].

## 5. Conclusions

Natural grass cultivation (NG) optimizes the transport of photoassimilates from the leaves to fruits by regulating C–N metabolism in the source organ (leaf) and sink organ (fruit), ultimately enhancing fruit quality. Moreover, NG management improves NUE and reduces soil NO_3_^−^-N accumulation in the deep soil layers.

## Data Availability

The data presented in this study are available on request from the corresponding author. The data are not publicly available due to later research needs.

## Supplementary Materials

The following supporting information can be downloaded at: https://www.mdpi.com/article/10.3390/metabo13080925/s1, Table S1: qRT-PCR primers.
