# Supplementary material for: Natural Grass Cultivation Management Improves Apple Fruit Quality by Regulating Soil Mineral Nitrogen Content and Carbon–Nitrogen Metabolism
Source: Metabolites. 2023 Aug 8;13(8):925. doi: 10.3390/metabo13080925 (PMC10456723; doi:10.3390/metabo13080925)
Supplement: Supplementary file 1 [file metabolites-13-00925-s001.zip › metabolites-2504983-supplementary.pdf]

Table S1 Primers used for qRT-PCR.

Gene name	Primer-Forward	Primer-Reverse
MdSOT1	5′- GACAGAACCTCAGACTCCAAAG -3′	5′- TGACCAGAGACCTGAACGATA -3′
MdSOT2	5′- CGTATCCAACTATGCCTTCTCC -3′	5′- GACACCGACGGCAAGAATAA -3′
MdSOT3	5′- GCCGGTACTCTAAACATCTACTC -3′	5′- GAAGATGACTCCCGCAAGAA -3′
MdSUT1	5′- GTGGTAATTTACCGGCATTTGTCG -3′	5′- AAGCTAGAGGCCGTAGGGCAAG -3′
MdSUT2	5′- CAGATGGTTTCCTTTCTTGTTGAG -3′	5′- TGCTGATACATAGTGAGTGGAACCT -3′
MdSUT4	5′- TGATTTACCTCCAGTTGGCATTG -3′	5′- GCCAAGTCCCAAAGACTCAATTC -3′
β-actin	5′- TGGTGTCATGGTTGGTATGG -3′	5′- CCGTGCTCAATGGGATACTT -3′
